# Recent Advances in the Surface Functionalization of Nanomaterials for Antimicrobial Applications

**DOI:** 10.3390/ma14226932

**Published:** 2021-11-16

**Authors:** Shahin Shah Khan, Irfan Ullah, Sadeeq Ullah, Ruipeng An, Haijun Xu, Kaili Nie, Chaoyong Liu, Luo Liu

**Affiliations:** College of Life Science and Technology, Beijing University of Chemical Technology, Beijing 100029, China; khan@mail.buct.edu.cn (S.S.K.); irfan@mail.buct.edu.cn (I.U.); 2018420012@mail.buct.edu.cn (S.U.); 18613228193@163.com (R.A.); hjxu@mail.buct.edu.cn (H.X.); niekl@mail.buct.edu.cn (K.N.); chaoyongliu@mail.buct.edu.cn (C.L.)

**Keywords:** nanoparticles, surface functionalization, NIR, MDR, antibacterial activities, nosocomial diseases

## Abstract

Innovations in nanotechnology have had an immense impact on medicine, such as in drug delivery, tissue engineering, and medical devices that combat different pathogens. The pathogens that may cause biofilm-associated nosocomial diseases are multidrug-resistant (MDR) bacteria, such as *Escherichia coli* (*E. coli*), *Pseudomonas aeruginosa* (*P. aeruginosa*), *Staphylococcus aureus* (*S. aureus*), including both Gram-positive and Gram-negative bacterial species. About 65–80% of infections are caused by biofilm-associated pathogens creating a move in the international community toward developing antimicrobial therapies to eliminate such pathogenic infections. Several nanomaterials (NMs) have been discovered and significantly employed in various antipathogenic therapies. These NMs have unique properties of singlet oxygen production, high absorption of near-infrared irradiation, and reasonable conversion of light to heat. In this review, functionalized NPs that combat different pathogenic infections are introduced. This review highlights NMs that combat infections caused by multidrug-resistant (MDR) and other pathogenic microorganisms. It also highlights the biomedical application of NPs with regard to antipathogenic activities.

## 1. Introduction

The use of medical devices in the field of medicine for therapeutic and diagnostic purposes has high importance. However, the risks associated with their usage are not negligible, particularly when these devices are intended to be inserted inside the body for long periods of time. The most important associated problems are colonization of bacteria on medical devices after surgeries, having a significant impact on patient health and the costs associated with the treatment of infectious disease. These barriers are commonly connected with bacteria that cause contamination of surgical wounds during surgery or during the time of hospitalization, which may result in a nosocomial infection [1]. So, these infections are extremely difficult to handle as bacteria (e.g., methicillin-resistant *Staphylococcus aureus* (MRSA) and vancomycin-resistant *Enterococcus* (VRE)) are more resistant to classical antibiotic therapies; particularly, medical devices, when colonized by bacteria, create a biofilm that is more dangerous for patient health [2]. Once bacteria make a coat on a hard surface, they can protect themselves by producing an extracellular matrix rich in exopolysaccharides. The bacteria colonization of the surface of medical devices or hard surfaces confer to biofilm a surface that provides a polysaccharide shelter that is resistant to our immune system [3].

The slow growth rate of bacteria and the biofilm microenvironment act together to facilitate the development of antibiotic resistance [4]. Furthermore, these infections are mainly caused by multidrug-resistant (MDR) bacteria, *P. aeruginosa*, *E. coli*, *S. aureus*, MRSA, VRE, *Acinetobacter baumanii*, and coagulase-negative *Staphylococcus*, which infect more than two million people annually in the United States, resulting in a significant loss of life and limb, with treatment requiring prolonged and costly therapeutic regimens [5]. The national institute of health (NIH) revealed that 65–80% of the infections are due to biofilm-associated pathogens [6]. The high risk regarding biofilm-associated nosocomial infections requires an international scientific movement to develop antimicrobial therapies to eliminate these infections [7]. For this purpose, nanotechnology is an emerging field in which different nanomaterials (NMs) act by surface plasmon resonance. Surface plasmon resonance is a powerful technique that retrieves information on the optical properties of NMs by striking incident light on its structure, which fluctuates the electrons at a specific resonance frequency and acts as a sensor to the transduced absorption band in the UV spectrum [8,9]. Furthermore, NPs can absorb near-infrared radiation (NIR), convert light to heat, and generate radicals, which highlight their therapeutic application against different pathogens [10]. These NPs are generally classified based on morphology, dimensionality, and chemical nature, and are categorized as isometric and inhomogeneous or dispersed and agglomerate [11].

This review highlights surface functionalization regarding the interaction of NPs with microbial cells, particularly how the unique characteristics of plasmon-based NPs influence their uptake pathway, NPs intracellular location and translocation, cytotoxicity, and biological application at the cellular level. The current knowledge of the physicochemical properties of NPs in light of antimicrobial activities will alleviate the effects of nosocomial diseases and combat these pathogens with their synergistic effects. These NPs combat microbial infection by mechanisms of reactive oxygen species (ROS)-based photodynamic therapy (PDT) and hypothermia-based photothermal therapy (PTT). PDT involves combating pathogenic bacteria via ROS, including superoxide anion (O_2_^−^), hydrogen peroxide (H_2_O_2_), singlet oxygen (^1^O_2_), and hydroxyl radical (⋅OH) [12]. It is also widely accepted that ROS can bind and damage the cell membrane and cell wall, thus destroying the defense system of pathogenic microorganisms [13,14]. The PTT mechanism of NPs involves the conversion of light into heat, which can kill pathogens by absorbing light (NIR) and converting it into heat [15,16]. Most importantly, we must construct safe and engineered NMs against different microbial infections and explore their biomedical applications.

## 2. Surface Functionalization and Characteristics of NPs for Antimicrobial Activities

To impart antimicrobial activity to NMs, there are three main different strategies. The first is to functionalize the NMs or polymers using antimicrobial agents such as quaternary ammonium compounds (QACs) [17,18] through a mechanism of contact killing. This strategy has been used extensively for the improvement in the microbicidal effectiveness of various surfaces. In particular, QACs are mostly used as disinfectants and microbicidal agents, having long alkyl chains to induce strong toxicity against amoeba and fungi, as well as to envelop viruses [19,20]. The second is to fabricate nanocomposites by adding filler such as metal-based NMs, e.g., Ag NPs. Four steps are necessary to create the antimicrobial activity of Ag NPs: (1) approach the surface of bacteria; (2) disrupt the cell wall and membrane; (3) produce free radicals and ROS, which exerts toxicity and oxidative stress effects; and (4) modulate signal transduction pathways. The third is the encapsulation of antimicrobial drugs or biomolecules, e.g., gentamicin. These functionalized NMs have strong toxicity against different infections caused by MDR bacteria, fungi, and viruses [21,22].

Different techniques are used for surface functionalization of carbon materials, i.e., oxidative process and non-destructive surface modification. In oxidative processes, the oxidized NMs further act as a precursor for chemical modifications such as silanization, polymer grafting, esterification, alkylation, arylation, etc., while non-destructive surface modifications are achieved by polymers wrapping, surfactant adsorption, and encapsulating processes [23,24]. Furthermore, the main aims of NPs surface functionalization are to tackle toxicity, the clearance of immune responses, and attachment of more complex and specific ligands to enhance efficacy and specificity for purposes such as antipathogenic activities through NPs [25].

Recently, the structural and behavioral changes of microorganisms have rapidly increased. The appearance of resistant bacteria by enhancing antibiotic adaptation is a significant concern for public health; thus, many efforts are needed to promote the efficiency and efficacy of antimicrobial substances. Surface functionalization of antimicrobial NPs can improve their efficiency, and various studies have shown their potential for use against resistant bacteria [26]; it also reduces the tendency to agglomerate carbon materials and increase the interfacial adhesion of carbon–polymer through covalent or ionic bonds [27].

For the use of NMs in biomedical applications, two main characteristics must be considered: toxicity and cellular uptake [28]. In nanotechnology, biocompatibility is one of the important characteristics of NPs for biomedical purposes, which describes the properties of materials being compatible with living tissues. The most important goal is to internalize NPs to targeted cells/tissues compared to non-targeted cells and to minimize the toxicity of the NPs into cells [29,30]. NPs also have unique physical and chemical properties due to their high surface area and nanoscale size [31].

## 3. Antimicrobial Activities of Nanomaterials

Antimicrobial resistance (AMR) occurs when bacteria, viruses, fungi, and parasites change over time and no longer respond to medicines, making infections harder to treat and increasing the risk of disease spread, severe illness, and death. It was implicated that more than 70% of micro-organisms causing infections are resistant to one or more antimicrobial agents used as treatment to eradicate infections [32]. Considering the current situation worldwide, there is concern about controlling MDR pathogens for infectious diseases preventions. Additionally, the significant prevalence of cross-contaminations and drug overuses, which may lead to biofilm development, may cause nosocomial disease that shows more resistance to standard therapies [33]. There are several mechanisms through which antibiotic resistance is achieved in bacteria, i.e., drug penetration prevention into a cell, antibiotics target changes, enzymatic inactivation of antibiotics, and active excretion of an antibiotic from a cell [34]. Some pathogenic fungal strains are also involved in biofilm formation and are more dangerous than bacterial biofilm. These fungal strains are likely yeast and filamentous fungi, and the most studied model is *Candida albicans (C. albicans)*. There are also other species (spp.), such as *Histoplasma capsulatum*, *Paracoccidioides*, *Trichophyton*, *C. glabrata*, *C. tropicalis*, *C. parapsilosis*, *Cryptococcus* spp., *Malassezia* spp., *Trichosporon* spp., *Fusarium* spp., *Scedosporium* spp., *Lomentospora prolificans,* and *Coccidioides* spp. Table 1 reveals the NMs that have activities against different MDR pathogens, antibacterials, antifungals, and antivirals that combat the biofilm-associated nosocomial pathogens mentioned above [35,36,37,38,39,40,41,42,43,44,45,46,47,48,49,50,51,52,53,54,55,56,57,58,59,60,61,62,63,64,65,66,67,68,69,70,71,72,73].

In this regard, developing non-conventional antimicrobial agents to prevent the aforementioned causes is under study. The rapid development of nanoscience and nanotechnology has shown promising potential for developing novel biocidal agents that would integrate with a biomaterial to prevent bacterial colonization and biofilm formation [33,90]. Metals with inherent antimicrobial properties such as silver, copper, and zinc, on the nanoscale, constitute a special class of antimicrobials that have a broad-spectrum antimicrobial nature and pose minimum toxicity to humans. Furthermore, a wide range of metals has antimicrobial activity, e.g., Ag, Al, As, Cd, Co, Cr, Cu, Fe, Ga, Hg, Mo, Mn, Ni, Pb, Sb, Te, and Zn [33,35,74,76,77,78,79,80,81,82,83,84,85,86]. These NMs have vast antipathogenic activities and are used to eradicate such pathogens that have strong resistance against normal therapies. These NPs tackle pathogens by having biocompatibility, biodegradability, reactive oxygen species (ROS) production, high absorption of NIR irradiation, and photodynamic and photothermal-conversion mechanisms, which show high performance in antipathogenic therapies. 

Understanding the main role of nanotechnology and its biomedical applications, in addition to antipathogenic therapies, is also promising; the most remarkable roles of NMs are in stem-cell-based therapies, neurodegenerative diseases, anticancer treatment, and gene delivery, which are currently being researched [91]. However, there are also a few drawbacks to be considered during the use of NPs for in vivo models. Most NPs disturb cell viability, alter mitochondrial functions, increase oxidative stresses, and alter tight junction protein expression of the blood barriers [92]. Hence, novel biomaterials that inhibit microbial growth and have low toxicity would be of great significance [33].

## 4. Outlook and Further Perspectives

Many scientists have reported several studies on NPs and their biomedical applications according to current demands. The biomedical application of NPs remains the most attractive aspect for scientists to improve clinical outcomes. The targeted drug and gene delivery diagnostic algorithmic treatment approaches improve efficacy while reducing side effects. However, the promising role of nanotechnology in combating different pathogens is more reliable and technical compared to standard antibiotics therapies. Metal NPs, owing to their unique adaptable physical and chemical properties, have been investigated widely in biomedical research.

Metal NPs have a unique interaction with light, which provides competent means for tracking nano-complex therapeutics carriers within the body, allowing more efficient therapies with low adverse effects compared to current conventional therapies. Interestingly, due to their high drug payload, surface chemistry, electrostatic charges, and photothermal behavior, metal NPs enhance therapeutics’ antimicrobial drug efficacy.

In this review, we discuss the promising role of functionalizing NPs with an antimicrobial agent, adding filler on its surface or encapsulating the antimicrobial agent, which allows for combating activities against different pathogens such as MDR, bacterial, fungal, and viral infections. Conclusively, based on the current literature on pre-clinical trials, plasmon-based metal NPs and their combination approaches are good prospects in microbial infectious therapies; for instance, biocompatibility, generation of singlet oxygen, and photothermal conversion properties of plasmon-based metal NPs are useful, and helpful in anticancer therapies. Further extensive research on NPs into their long-term toxicity is essential for successful clinical use in biomedical applications. We also think that the development of simple and low-cost inorganic antimicrobial agents such as NPs as alternatives for traditional antimicrobial agents might be promising for the future of the pharmaceutical, food, and medical industries.

## Figures and Tables

**Table 1:** Activities of different NPs against MDR-pathogens, antibacterial, antifungal and antiviral.

NPs	Activity	Targeted Pathogen	Refs.
Au NPs	MDR pathogens	Methicillin-resistant *S. Aureus,* various groups of MDR (multidrug-resistant) Gram-positive (MRSA, MRSE, and MLSB), and Gram-negative (extended-spectrum betalactamase (ESBL), AmpC, and CR) pathogens	[32,74]
Ag NPs		*S. epidermidis,* MRSA, VRE, ESBL-producing organisms, MDR *E. coli, P. aeruginosa, K. pneumoniae,* carbapenem- and polymyxin B-resistant *A. baumannii, Salmonella typhi (S. typhi),* and *S. aureus* Carbapenem-resistant *P. aeruginosa* and carbapenem-resistant *Enterobacteriaceae (CRE)*	[32,75,76,77,78,79]
Cu NPs		*S. aureus, E. faecalis, E. coli,* and *P. aeruginosa, K. quasipneumoniae* and *Enterobacter* sp.	[80,81]
Se NPs		*S. aureus, P. aeruginosa,* and *E. coli*	[82]
Al NPs		MDR *K. pneumoniae*	[83]
Metal oxide NPs ZnO_2_-NPs ZnO NPs TiO_2_ NPs		MDR *E. coli, S*. *aureus*	[84,85]
CeO_2_ NPs		*K. pneumoniae*	[86]
Antibacterial activities of different NPs			
Ag NPs	Antibacterial	*E. coli, B. subtilis, S. aureus,* methicillin-resistant coagulase-negative *Staphylococci,* VRE *faecium,* ESBL-positive *K. Pneumonia, S. typhi, Vibrio cholera*	[36,37,38,39]
Au NPs		MRSA, VRE*-faecium, E. coli, P. aeruginosa*	[40]
TiO_2_ NPs		*E. coli 0157:H7, S. aureus,* *L. monocytogenes S. enteritidis,* *P. fluorescens*	[41]
ZnO NPs		*E. coli 0157:H7, B. subtilis, P. fluorescens,* *L. monocytogenes, S. enteritidis, S. aureus,* *S. typhimurium*	[42,43]
CuO NPs		*B. subtilis, L. monocytogenes, S. aureus,* *E. coli*	[44]
HSA-GO-Pd		*E. coli*	[87]
MgO NPs		*B. subtilis, E. coli, S. aureus, B. megaterium*	[45,46]
CaO NPs		*S. aureus, S. epidermidis, E. coli, S. mutans*	[47]
Al_2_O_3_ NPs		*E. coli, P. aeruginosa, S. aureus, B. subtilis,* *K. aerogenes, P. desmolyticum*	[48,49,50]
SiO_2_ NPs		*E. coli, S. mutans, B. subtilis*	[51]
Clay NPs		*E. coli, E. faecalis, S. aureus,* *P. aeruginosa*	[52]
Antifungal activities of different NPs			
Ag NPs	Antifungal	*C. albicans,* *T. mentagrophyts, B. sorokiniana, M. grisea*	[53,54,55]
ZnO NPs		*B. cinerea, P. expansum, A. flavus, S. cerevisiae, C. albicans, R. stolonifera, F. oxysporum, Mucor*, *A. fumigatus*, *A. niger*, and *F. solani*	[50,54,55,56]
TiO_2_ NPs		*Candida.* spp. *P. Expansum, A. niger* spp. *P. oxalicum*	[57,58]
CuO NPs		*A. niger, Rhizopus oryzae, A. flavus,* *Cladosporium carrionii, Mucor,* *S. cerevisiae, P. notatum, C. albicans*	[59,60]
MgO NPs		*Saccharomyces cerevisiae, C. albicans,* *A. niger, R. stolonifer, Fusarium oxysporum f.* sp. *lycopersici*	[61,62]
CaO NPs		*S. cerevisiae, C. albicans, A. Niger, R. stolonifer, C. brevisporum*	[61,63]
Au NPs		*Puccinia graminis tritci, A. flavus, A. niger C. albicans*	[64,65]
SiO_2_ NPs		*Candida.* spp., *Dermatophytes* spp., *A. niger,* and *S. racemosum*	[66,67]
Al_2_O_3_ NPs		*Candida.* spp., *S. quadricauda, A. niger, A. flavus, Fusarium* spp., and *Alternaria* spp.	[66,68]
Antiviral activities of different NPs			
Au NPs	Antiviral	HIV virus, Influenza virus, Herpes Simplex virus (HSV-1)	[69,70,71]
Ag NPs, Ag_2_O|AgO-NPs		HIV-1, Influenza virus, Herpes Simplex virus, Respiratory syncytial virus, Monkey pox virus, SARS-CoV-2	[69,70,71,72,88,89]
TiO_2_ NPs		Inactivates bacteriophages	[50]
CuI NPs		Influenza A virus, feline Calicivirus (FCV)	[73]

## Data Availability

The data presented in this study are avalible on request from the corresponding author.
